# Medical student support for vulnerable patients during COVID-19 – a convergent mixed-methods study

**DOI:** 10.1186/s12909-020-02305-z

**Published:** 2020-10-22

**Authors:** Tirion Hughes, Eleanor Beard, Amelia Bowman, Joyce Chan, Katrina Gadsby, Martha Hughes, Maya Humphries, Aaron Johnston, Georgina King, Megan Knock, Kaveeta Malhi, Gerda Mickute, Ebubechi Okpalugo, Madeleine Oliver, Vimukthi Perera, Florence Pickles, Lily Pollock, Lucienne Pullen, Ffion Samuels, Harriet Sexton, Laura Shutler, Rebecca Smith, Pippa Tanner, Emma Ladds

**Affiliations:** 1 Oxford Medical School, Medical Sciences Division, University of Oxford, Wellington Square, Oxford, OX1 2JD UK; 2 Nuffield Department of Primary Care Health Sciences, University of Oxford, Observatory Quarter, Woodstock Road, Oxford, OX2 3GG UK

**Keywords:** COVID-19, Medical students, General practice, Education

## Abstract

**Background:**

The coronavirus pandemic has exerted significant impacts on primary care, causing rapid digital transformation, exacerbating social isolation, and disrupting medical student and General Practice [GP] trainee education. Here we report on a medical student telephone initiative set-up by a final year GP trainee (the equivalent of a family medicine resident), which aimed to support patients at high risk and vulnerable to the Coronavirus Disease of 2019 [Covid-19]. In addition, it was hoped the project would mitigate a digital divide, enable proactive anticipatory planning, and provide an active learning environment to compensate for the pandemic’s impact on medical education.

**Methods:**

Thirty-three medical students conducted daily telephone conversations with high risk and vulnerable patients as specified by the initial NHSE published lists. They confirmed public health messages, offered details for voluntary support groups, established need for medication delivery, explored levels of digital connectivity, and prompted discussions around end-of-life choices. Students had access to online reflective resources and daily remote debriefing sessions with the GP trainee.

A convergent mixed-methods evaluation was subsequently undertaken, using quantitative process and descriptive data and individual qualitative interviews were conducted according to a maximal variation sampling strategy with students, General Practitioners [GPs], and the GP trainee. Inductive thematic analysis was then applied with cross-validation, respondent validation, and rich evidential illustration aiding integrity.

**Results:**

Ninety-seven ‘high risk’ and 781 ‘vulnerable’ calls were made. Individuals were generally aware of public heath information, but some struggled to interpret and apply it within their own lives. Therefore respondents felt students provided additional practical and psychological benefits, particularly with regard to strengthening the links with the community voluntary groups. The project was widely liked by students who reported high levels of skill development and widened awareness, particularly valuing the active learning environment and reflective feedback sessions.

**Conclusion:**

This study demonstrates utilization of medical students as wider assets within the primary health care team, with an initiative that enables support for vulnerable patients whilst promoting active medical education. Ongoing integration of students within ‘normal’ primary health care roles, such as chronic disease or mental health reviews, could provide similar opportunities for supported active and reflective learning.

**Supplementary information:**

**Supplementary information** accompanies this paper at 10.1186/s12909-020-02305-z.

## Background

*“Honestly, if you want to be a doctor then you’ll want to be doing this. Learning wise it’s very useful, medicine is a lot of communication and yeah, it’s a great scheme.” (1*^*st*^ *year Graduate Entry Student [1*^*st*^ *yr GES])*

The role of the ‘family physician’ has always been grounded within the community. However, in recent years, collaboration between primary care teams, community services, private organisations, and the voluntary sector, alongside patients and families has become increasingly important. Within this framework, General Practitioners (GPs) may also be viewed as organisations, interacting and reacting as components within a complex system [1].

The COVID-19 pandemic has transformed these interactions. Not only have GPs had to ensure accurate communication of public health messages, develop or adapt relationships with the voluntary sector, and protect healthcare system capacity though proactive anticipatory care planning – whilst also maintaining some continuity of care – but they have done so whilst transitioning to a comprehensive digital practice [2].

The impact on medical student and GP training has been similarly disruptive [3, 4]. Acknowledging the tensions between safeguarding education and maintaining the frontline workforce, many medical schools and Vocational Training Schemes have cancelled or postponed training opportunities, impeding trainee progression and heightening anxiety.

These individual and system challenges are not insignificant. However, medical students, GP trainees, and the primary care teams and communities they represent, have numerous assets, and collective experience. In accordance with Asset Based Community Development theory [5], connecting, empowering and activating these individuals could both support a response to COVID-19, whilst also fostering longer-term integration between students, primary care teams, and local communities.

The initiative presented in this paper reflects these aims. It recognizes and utilizes the skills of medical students, supporting them in providing telephone contact to patients initially categorized by NHSE as ‘high risk’ and ‘vulnerable’ to COVID-19 infection.

The calls ensured awareness of relevant public health messages and provided details of local community groups. Need for medication delivery and levels of digital connectivity were assessed and reported to the GP practice, enabling personalised communication and patient care, and patients and families were also prompted to consider end-of-life-issues. Receptive patients were highlighted to the usual GP for formal care planning conversations.

Recognising the pedagogical value of active learning proposed by Prince [6], this intervention also supported GP and medical student training, encouraging development of leadership, education, communication, and consultation skills.

Finally students were supported in conducting a mixed-methods analysis to explore compliance with the aims and assess the experiences of those involved. In addition to the reflective activities undertaken throughout, this promoted Prince’s practice of *‘doing meaningful learning activities’* and *‘thinking about what [you] are doing*’ [6], combined with the higher components of Bloom’s Taxonomy of Educational Objectives – analysis, synthesis, and evaluation [7] – thus maximizing educational value.

This paper attempts to provide a mixed-methods evaluation of this service improvement project.

## Methods

### The student-support initiative

Any pre-clinical or clinical student at Oxford Medical School recruited by a final year GP trainee through college medical societies, social media groups, and by word-of-mouth could volunteer to participate.

An initial briefing introduced students to the rural West Oxfordshire GP practice, its population, and the project aims. These were:

1. To ensure all COVID-19 high risk patients and those aged 70 or over in the vulnerable category (identified by National Health Service England [NHSE] on 23/3/20) were aware of relevant Public Health England advice. Care home residents were excluded [8].
2. To ensure patients could collect medications, had community support group contact details, and assess levels of digital connectivity;
3. To prompt consideration of end-of-life care issues and record whether a follow-up call with a GP would be appropriate (this was added one week after the start for students who felt comfortable);
4. To provide a supportive active learning environment for medical student and GP trainee development.

An information sheet containing conversation prompts, contact details for the GP trainee supervisor, and online reflective log were provided.

The initiative took place between 26/3/2020 and 30/4/2020. Students chose up to 3 daily calls and could opt-out at any point. Data collected from the calls was collated and shared for follow-up with the relevant GP practice member.

All communications were held using Zoom, phone calls, WhatsApp, or email. In compliance with data protection regulation, student phone numbers were withheld and personal data was minimised and shared securely using Egress. No patient details were shared over Zoom or WhatsApp. If the student had clinical concerns, they discussed this with the GP trainee on the phone.

The GP trainee was always available to discuss clinical concerns or debrief any student. If it was necessary, the GP trainee then followed up with the patient. A random selection of patients were also called by the GP trainee to assess quality of student interactions.

Daily group debrief sessions facilitated discussion of positive and negative experiences, prompted reflection, provided consultation and communication skills theory, and practical suggestions for improvement.

### Quantitative analysis

Process data included: the number of eligible patients; total calls attempted; and failed communication attempts.

Information was captured electronically regarding medication delivery and digital connectivity. Where available, type of digital communication was also assessed. Calls detecting unaddressed health or medication concerns and those that included end-of-life care were analysed. An EMIS search identified patients with subsequent GP care planning consultations.

### Qualitative analysis

One student did not consent to participate due to pressure of other volunteering commitments. Of the remainder, a maximal variation sampling strategy, partially based on a pre-selection questionnaire (developed for this project and including: year of study, under−/postgraduate status, gender, previous experience talking to patients/conducting phone calls, and previous personal experience of COVID19), selected a sample of 13 students, the GP trainee and two GPs. All were invited to participate and had the opportunity to decline. Whilst data saturation was considered, pragmatically it was felt that this was a less important determinant of sample size in ensuring a timely evaluation.

Individual, remote semi-structured interviews of around 20–30 min duration were conducted and audiorecorded via Zoom with recorded verbal consent from interviewees. Interview guides are included in the supplementary data. A small group of female students with limited research experience (JC, GK, KM, EO, VP, FP, LPo, HS, and RS) conducted this phase of the study under the guidance of the NIHR-funded academic GP trainee who had previous research experience in conducting mixed-methods service evaluations. Some of the participants who were other medical students may have been known to the interviewers or from working together on the initiative and all were aware of the general reasons why the investigators were conducting the research. A number of factors with potential bias were considered for the different investigators, including previous research experience or personal experience of COVID-19. Cross-validation, respondent validation and rich use of primary evidence were used to help ensure these did not compromise integrity of data interpretation.

Loose interview guides were developed collectively by the team of qualitative student researchers. Questions explored participant expectations, positive and negative experiences, and subjective assessment of any impact on patients, the GP practice, local community and project participants. Due to the need for a rapid, pragmatic evaluation during the acute phase of the pandemic there was no pilot testing. No repeat interviews were carried out.

Interviews were transcribed and analysed thematically by the same student team using NVIVO12. The transcripts were not returned to the interviewees, but respondent validation of the identified themes was later used. The project aims were applied as a deductive coding framework to enable a level of evaluation. Students shared and discussed the coding tree in pairs and then collectively as a group.

This paper reports a quality improvement initiative and an associated mixed-methods service evaluation of the process and outcomes. Under current UK guidance formal ethical approval is therefore not required [9, 10].

## Results

### Quantitative

#### Process information

Thirty-three students took part, from first year preclinical to fifth year clinical and graduate entry students. The mode (36%) were in their fourth year (1st year of clinical studies).

The majority reported low-medium levels of previous patient interaction, whilst 2 reported almost none and 5 high levels. Around 10% had previous experience making ‘cold’ calls.

**Fig. 1:**
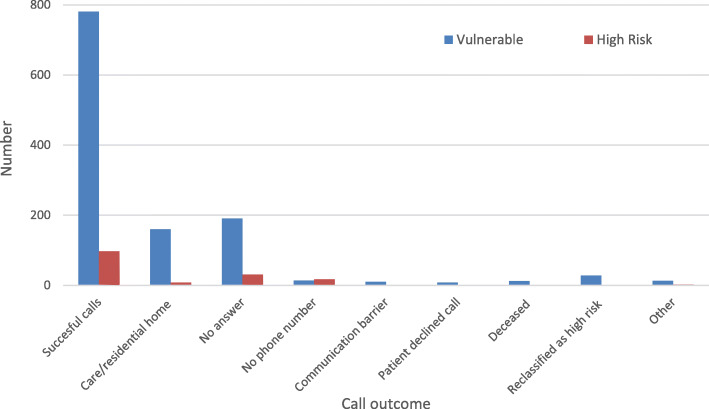
Breakdown of call attempts

One hundred fifty-six high risk and 1217 vulnerable patients were included, with average ages of 64 and 85 respectively. 97 ‘high risk’ and 781 ‘vulnerable’ calls were made successfully with Fig. 1 detailing failed attempts. The majority were exclusions for care home residents or unanswered calls (Table 1).

**Table 1:** Breakdown of call attempts and outcomes amongst the high risk and vulnerable patients

	High Risk *n* = 156 (%)	Vulnerable *n* = 1217 (%)
**Number of total calls**	97 (62.2)	781 (64.2)
**Care Home Residents** (therefore excluded)	8 (5.1)	161 (13.2)
**Deceased**	0	12 (1.0)
**Reclassified as high risk**	N/A	27 (2.2)
**Unable to contact or collect information**	51 (32.7)	236 (19.4)
**Able to collect medication**^a^	85 (87.6)	695 (88.9)
**Digitally connected**^a^ (mobile phone/internet or both)	75 (77.3)	437 (55.9)
**End-of-life discussions**^b^	N/A	
Existing care plan	N/A	41 (28.2)
Wished to discuss further	N/A	28 (19.3)
Strongly against advanced decisions	N/A	29 (20.0)
Inconclusive	N/A	47 (32.4)

^a^Denominator is number of successful calls (*n* = 97 high risk; *n* = 781 vulnerable)

^b^End-of-life discussions were introduced later into the conversations after all the high-risk calls were complete in response to further guidance from NHSE *n* = 145

(Percentage totals are the result of rounding)

#### Medication delivery and digital connectivity

The majority of both high risk and vulnerable respondents could organise medication collection – 88 and 89% respectively. The remainder either had no capacity to do so or relied on community support groups.

Students found it challenging to collect data regarding digital connectivity. Whilst every patient had a landline, 22% of high risk and 44% of vulnerable respondents reported some impairment of connectivity i.e. either not making regular use of mobile phone, internet (and email) services, or both (Table 1).

#### End-of-life care

Optional prompting around end-of-life care was introduced to the project after the conclusion of the high-risk calls. Data are therefore only available for 145 vulnerable patients.

Of those, 28% of respondents either had an existing care plan with a resuscitation decision or were open to further exploration. 19% expressed a desire for further discussions with a GP. In contrast, 20% reported strong views against resuscitation decisions, and a third of conversations were inconclusive. 6% of the 76 EMIS-coded resuscitation discussions that took place over the subsequent 9 weeks were for patients following student discussions (Table 1).

#### Other medical issues

Just over 10% of vulnerable respondents raised some other query around medications, prescriptions or appointments. Many viewed potential system overburdening as a barrier to not consulting the GP practice or secondary care service.

### Qualitative

#### Communication of public health messages and community support information

Respondents were generally aware of PHE guidance despite progressive disengagement with media sources throughout the project. However, many found it challenging to interpret the relevance of the advice and consider practical application within their own lives. For example, one student reported,

*“many patients didn’t realise they were in the vulnerable group and they’d say, “Oh no I’m not.” Then they’d ask what makes someone vulnerable.’ (2*^*nd*^ *yr)*

Thus giving tangible patient-centred advice,

*“there were a lot of them that didn’t know the advice was to call 111 if they became unwell”, (1*^*st*^ *yr GE)*

clarifying practical details,

*“Questions would be like should I be wiping my post or is it ok if I see a relative who is also isolating. Questions that aren’t as well covered over TV.” (5*^*th*^ *yr)*

or helping plan for hypothetical scenarios,

*“they thought they were sorted but actually they didn’t have a contingency plan for what would happen if say their husband were to become unwell - who would pick up your medication if everyone in your house were to have to self-isolate for fourteen days for example” (1*^*st*^ *yr GE)*

were perceived by students as particularly beneficial for patients.

Awareness of the community support groups was similarly varied. However patients particularly valued this information given its potential tangible benefits. One reported,

*“About 50% of people I spoke to didn’t have support group contacts and wanted them. For example, one lady wasn’t getting any fresh fruit and vegetables because she was depending on a local village store and didn’t know about the support groups that could help her” (1*^*st*^ *yr)*

whilst another highlighted the psychological reassurance of the community ‘safety net’,

*“even if now they have relatives collecting the shopping, they were appreciative that the support groups can be a good backup for if that person becomes unwell” (5*^*th*^ *yr)*

#### Medication collection and other health concerns

Whilst patients appreciated the concern around their medications, many raised other clinical or administrative concerns about which they were reluctant to trouble the practice*.* Students clearly valued their role in ameliorating these concerns,

“*I’m doing something that is benefiting patients and as the GPs are so overstretched, it’s not really a service they would realistically be able to provide if they didn’t have student volunteers.” (4*^*th*^ *yr)*

and the practical utility of mitigating the pandemic’s impact on continuity of care was emphasized by the GP trainee,

*“It’s been helpful because it’s demonstrated the impact of COVID on continuity of our standard care. Normally we’d be trying to manage a lot of chronic disease, a lot of hypertension, diabetes, COPD and asthma. A lot of the calls have picked up issues where that continuity of care has been disrupted. Sometimes I can’t do anything about it but sometimes it’s really important it’s followed up.” (GP trainee)*

#### End-of-life care discussions

All students expressed anxiety about discussing end-of-life issues. Whilst those in earlier years of study emphasized the sensitivity and were concerned about their lack of experience or student status and therefore potential impact on the patient,

*“it was potentially more sensitive conversations or vulnerable people, kind of choosing the right words for this was definitely a bit anxiety provoking” (1*^*st*^ *yr GE)*

those in later years were concerned how the remote nature of the conversations differed from the ‘ideal’ way in which they had been taught to conduct them. One fifth year reflected,

“*We’d done the communications skills sessions and had all the lectures. That was really different though because the idea of doing it over the phone would be top on the ‘don’t do it this way’ list and calling someone you’d never met before.” (5*^*th*^ *yr)*

Whilst negative experiences were reported, many related to the challenge of remote conversations, although some reflected communication inexperience. However, the majority of experiences were positive, contrasting with prior expectations. This likely reflected the discretion given to students about the appropriateness of introducing the subject. As one reflected,

*“The ones that I asked were all receptive because I didn’t ask the ones that didn’t want to talk to me. About half had already discussed it with their GP or had decided mostly they didn’t want to be resuscitated. The other half, because I explained that it was something we were asking all the patients, were happy to talk about it and made jokes.” (1*^*st*^ *yr GE)*

GPs and the GP trainee felt it appropriate and beneficial that students discussed end-of-life issues in this context. However, they expressed concern for student wellbeing and ensuring they had adequate support,

*“I was conscious that you’re [students] not around collectively in Oxford so it’s not like we can just pop down to the pub for a drink or something to discuss everything. So I was more worried about whether we were going to be able to give adequate support to students who were not used to having those kind of conversations” (GP trainee)*

#### Providing a supportive learning environment for GP and medical student training

All trainees recognized the disruption to their education and wider lives. The majority of students discussed their return home following the postponement of formal teaching whilst the GP trainee reflected on the *‘upheaval in systems, and normal working process [within Primary Care]’* impacting general practice training.

Potential learning was not widely viewed as a motivating factor for participation. Rather students reported a desire to contribute despite their limited experience and a need to fill free time,

*“I just didn’t want to feel useless I think. I don’t know about what your year are going through, but our core exam was pushed to September and then it was still being approved and then our ethics and things are going to be open book and everything was just sort of chucked in the air. And meanwhile, I just got this saying ‘would you like to do this?’ and I think it was for me a very welcome project for feeling useful, feeling like we could be of any help at all with our limited experience at the moment because it feels like we are almost a burden more than we are help, you know?” (1*^*st*^ *yr GE)*

Most students felt their communication had improved, particularly around exploring expectations, tailoring language, using silence, transference, chunking, and sense-checking. Other reported benefits included exposure to clinical topics, learning when to admit you do not know, and gaining a greater awareness of the broader context of medicine and social determinants of health,

*“[participating] reminded me of the wider context of medicine, and understanding people’s social situation, their life situation, and realising that sort of little things like communicating with your friends and your family are so much a part of health as any type of medication or disease” (4*^*th*^ *yr)*

For students with limited previous patient interactions, there was value in the active ‘doing’ of the project, whereas those in later years emphasized the added utility of real patients over simulated scenarios and the challenge of moving beyond ‘textbook communication’,

*“we have communication/skills, which are kind of where they bring in actors and things like that, but I think it’s still very different from when you’re doing it to actual patients. And I think one of the things that I struggled with is when they’re telling you something which is a bit sad or a bit sensitive the response you give to it. I think one of the textbook things to say is ‘I’m really sorry to hear that’ but it doesn’t actually work in all situations/scenarios and I think it was more learning to adapt and respond to people appropriately”( 4*^*th*^ *yr)*

The GP trainee also reflected how the active coordination of the project helped develop leadership skills for her wider roles within general practice,

*“there are all sorts of people involved in the primary care team and so much of what I do is about coordination. What’s been quite interesting trying to look after vulnerable patients is that I have to try and coordinate [students], I have to try and coordinate what the community volunteer teams are doing to a certain extent, a little bit around the medical care for these people and to try and plan strategically how we would cope with things like end-of-life care if that became a large scale issue. I think probably over the project I’ve learnt how better to coordinate all those different things”(GP trainee)*

All respondents valued the debriefing sessions, emphasizing the learning potential from feedback, discussion and reflection, but also the social connectivity, which mitigated some of the isolation students were experiencing. Students were also reassured by the prompt responses from the GP trainee if they raised concerns, with some reporting surprise at the level of support offered. This made them feel valued,

*“I was pleasantly surprised at how committed [the GP trainee] was to prioritising our Zoom meetings every day […] and I thought it was really, I don’t know what the word is, but I was really happy that she was willing to do that for us in spite of everything.” (1*^*st*^ *yr GE)*

In contrast, the GP trainee recognized the tacit support offered by her colleagues, but also valued the opportunity for independent development.

#### Impacts on participants, patients, and GP practice

All participants recognized that the greatest value to patients lay in the social connectivity and sense of self-worth that the calls represented. One student reflected on a bittersweet call,

*“I spoke to an elderly lady a few days ago. I was the first person she’d spoken to all day. She was very lonely, and we had a really long nice conversation about all sorts of random stuff […] It was a nice conversation but a bit sad to put the phone down. She sounded sad to put the phone down. She said about 10 times throughout the call she was so grateful for the call and it had perked her up” (1*^*st*^ *yr)*

whilst another reported the perceived emotional benefit relative to that generated by the wider media,

*“I think it’s the sense that they’re worth someone's time even though they’re old. They might have seen things on the news saying they might not get hospital care, so nice to see that they are worth someone's time. (5*^*th*^ *yr)*

Both the GPs and GP trainee recognized the reduction in overall practice workload thanks to the students’ input,

*“It has minimised [the workload], because since it started, the government told us that’s what we had to do but you’d already done it. If you hadn’t, we would have had to do it, ringing through the patients and offering them support.” (GP partner)*

and although it increased that of the GP trainee, this was deemed appropriate as trainees do not carry the same patient list burden as qualified GPs.

Whilst the students generally felt well supported, they also reported the personal impacts of the calls. For example, one,

*“[thought] about them quite a lot, especially the more emotive ones where you leave and it makes you really sad, like the lady I spoke about earlier who was on her own. I’ve been thinking about calls like that a lot.” (1*^*st*^ *yr)*

whilst another was more positive,

*“Because I’d make the calls in my bedroom and then go downstairs and my parents would ask how they went, and I would say ‘ they were good today’ or ‘I spoke to this nice lady’ so I’d reflect on them for a bit. When a phone call went really well, it sounds really sad, but I’d have this kind of fuzzy feeling for a while after.” (4th yr)*

See supplementary data 1 and 2 for student case studies.

## Discussion

### Summary

Medical students are a valuable, but often overlooked, members of the wider primary care team. In this intervention we demonstrate an effective application of Prince and Bloom’s principles of active and reflective learning to provide a dual-purpose intervention, which supports vulnerable patients in accordance with the principles of asset-based community development, whilst also mitigating the disruption of the COVID-19 pandemic on medical education.

### Strengths and limitations

This is an innovative attempt to recognize non-traditional assets within the wider primary care team and exploit potential opportunities offered by the COVID-19 pandemic to support vulnerable individuals, whilst also strengthening the links between primary care and the local communities, mitigating consequences of rapid digitalization, and compensating for disruption to formal medical training.

Given the confusion surrounding the classification of high-risk and vulnerable COVID-19 patients, this intervention only included those on the original NHSE lists published on 23/3/2020. Some individuals may later have been removed, whilst others will have been missed. Several individuals also passed away throughout the intervention without being removed from the list, resulting in potentially distressing conversations with family members.

Whilst a maximal variation sampling strategy was used, the size was pragmatically limited to avoid overloading student researchers. It is therefore possible that data saturation was not reached. Moreover, despite attempts to ensure transparency and validity of iterative qualitative interpretation including multiple coders, respondent validation, and rich use of illustrative quotes, there may be some deviation from the primary data.

### Comparison with existing literature

Patients are known to preferentially seek and trust health advice from physicians they know and respect [11], whilst previous pandemics have revealed general distrust of government advice [12]. As members of the local primary care team, students were able to prompt interpretation and individual application of the general health messages as well as provide practical information.

Given the well-known psychosocial determinants of health [13] and recent emphasis on social prescribing within primary care, the rapid surge of COVID-related volunteering initiatives [14] offers great potential to support vulnerable or isolated individuals. Using students to communicate such initiatives to patients strengthens the links between GPs and local communities, developing the potential for ongoing co-production of supportive interventions.

Similarly, the rapid COVID-enforced digital transformation of primary care risks heightening health inequalities imposed by a digital divide. Individuals aged over 75, with particular conditions, in specific socioeconomic groups or geographic locations have been shown to be at particular risk [15]. Respondents in this intervention, particularly the ‘older’ vulnerable category, did show significant reductions in digital connectivity. Collecting this information could facilitate tailoring of access, services, and communication options, thus mitigating the detrimental impacts of digital exclusion whilst enabling the plethora of potential population benefits.

Both students and patients expressed surprise that they were perceived as being worth someone’s time i.e. they were valued. In accordance with Maslow’s ‘need to belong’ [16] or the concept of ‘relational value’ proposed by Leary [17], reinforcement of this belief could be considered crucial both for educational self-development and in promoting acceptance around end-of-life issues.

Indeed, perceived violation of this value may have underpinned some of the concern around the *‘en masse’* resuscitation decisions imposed for patients in Brighton, Hove and Wales during the pandemic [18]. In contrast, this intervention demonstrated a personalised approach with students raising end-of-life care issues in a priming ‘pre-conversation’ – a technique associated with patient satisfaction and shown to improve concordance between patient goals and care received [19].

Whilst simulation scenarios are widely accepted by medical students [20], many prefer real-patient contact [21], particularly to contextualize learning [22] - a preference that was supported here. Moreover, both GP trainee and students valued the Zoom debrief calls, which encapsulated the higher aims of Bloom’s taxonomy of learning. They appreciated the discursive feedback and evaluative reflection, which deepened both communication and leadership skills as well as awareness of the broader context of medicine and life.

## Conclusions

This study illustrates that challenging clinical scenarios within primary care can provide valuable opportunities for the GP practice, local community, medical students, and trainees. The potential for student involvement in ‘regular’ general practice activities, including chronic disease or mental health reviews or initiatives with local communities, could represent excellent ongoing educational opportunities. These possibilities merit further exploration.

## Supplementary information

**Additional file 1.** Case Study 1. Personal case study of student participant in intervention.

**Additional file 2.** Case Study 2. Personal case study of student participant in intervention.

**Additional file 3.** COREQ checklist.

**Additional file 4.** Interview Questions.

## Data Availability

The datasets are not publicly available for this study because they contain patient identifiable information. For this reason they are also not available by personal request.

## Abbreviations

COVID-19: Coronavirus Disease of 2019
EMIS: Egton Medical Information Systems
GES: Graduate Entry Student
GP: General Practitioner
NHSE: National Health Service England
PHE: Public Health England
Yr: [Year]
