# Supplementary material for: Medical student support for vulnerable patients during COVID-19 – a convergent mixed-methods study
Source: BMC Med Educ. 2020 Oct 22;20:377. doi: 10.1186/s12909-020-02305-z (PMC7578590; doi:10.1186/s12909-020-02305-z)
Supplement: Supplementary file 1 — Additional file 1. Case Study 1. Personal case study of student participant in intervention. [file 12909_2020_2305_MOESM1_ESM.docx]

[Student One – first year preclinical student]

As a first-year medical student, this felt like a great opportunity to help-out in the COVID-19 crisis, whilst gaining some valuable experience talking to patients and improving my communication skills.

One memorable call was to an elderly lady living alone. After a general conversation, she talked about her career and related memories. She clearly appreciated the opportunity to talk about pre-COVID-19 times and her wonderful, interesting life experiences. She was well supported but confessed she was bored. We discussed my life as a medical student and my motivation for calling patients. Her response surprised me. She told me that patients will open up more to medical students than medical professionals because we don’t have the same status – we’re just like them. She told me how valuable we were and how much my call had brightened her afternoon.

This call was so encouraging because I could see the clear positive impact it had. Over the project I’ve learnt a lot and grown greatly in my ability to communicate with people. My only regret is choosing to just call one patient a day!
