# Supplementary material for: Medical student support for vulnerable patients during COVID-19 – a convergent mixed-methods study
Source: BMC Med Educ. 2020 Oct 22;20:377. doi: 10.1186/s12909-020-02305-z (PMC7578590; doi:10.1186/s12909-020-02305-z)
Supplement: Supplementary file 2 — Additional file 2. Case Study 2. Personal case study of student participant in intervention. [file 12909_2020_2305_MOESM2_ESM.docx]

[Student Two - first year preclinical student]

Despite being well supported and informed, I was initially very nervous about talking to patients. However, as the majority were clearly delighted to receive a call and were understanding if I couldn’t answer their questions, my confidence grew.

Daily group debriefing calls encouraged me to think critically about the day’s conversations. This emphasised the importance of self-reflection in refining communication skills for the best patient experience. Two illustrating calls stick in my mind.

The first involved a very positive end of life conversation with an elderly couple. I had soon realised that skirting around this topic was of little use. It was usually best to take a direct, less confusing approach. We discussed why some people might not want to go to hospital in the case of serious illness. They hadn’t thought about this but welcomed the opportunity to consider their preferences. I was left contemplating both how mortality is so avoided in our society and also how empowering it can be to be offered the chance to consider it.

The second conversation didn’t go well. The patient was anxious and questioned why I was bringing up such issues. I panicked and rushed the information, ending the call concerned that I had distressed her. I immediately contacted the supervising doctor, who followed up with the patient. Reassuringly she was not upset, just a bit confused. At the evening debrief we discussed the issue of transference in consultations – the impact the patient’s own thoughts and feelings have on you – and how difficult it can be sometimes to tell how a conversation has gone for another person. Finally, I had to accept that not all conversations will go well but there will be lessons from each that will make the next one better.

I am so glad I had the opportunity to take part. My initial motivation in signing up was to help provide reassurance to vulnerable patients and collect information for the GP practice. However, I actually feel the experience has been as useful for me as it has been for the GPs and their patients. These calls were my very first one-on-one conversations with patients, and will be interactions I shall never forget.
