# Supplementary material for: Medical student support for vulnerable patients during COVID-19 – a convergent mixed-methods study
Source: BMC Med Educ. 2020 Oct 22;20:377. doi: 10.1186/s12909-020-02305-z (PMC7578590; doi:10.1186/s12909-020-02305-z)
Supplement: Supplementary file 3 — Additional file 3. COREQ checklist. [file 12909_2020_2305_MOESM3_ESM.docx]

|  |  |  |
| --- | --- | --- |
| **Domain 1: Research team and reflexivity** |  |  |
| 1. Inter viewer/facilitator | Which author/s conducted the interview? | Methods  Page 8  Line 207 |
| 2. Credentials | What were the researcher’s credentials? | Methods  Page 8  Line 206-8 |
| 3. Occupation | What was their occupation at the time of the study? | Methods  Page 8  Line 206-8 |
| 4. Gender | Was the researcher male or female? | Methods  Page 8  Line 206 |
| 5. Experience and training | What experience or training did the researcher have? | Methods  Page 8  Line 206-8 |
| 6. Relationship with participants established | Was a relationship established prior to study commencement? | Methods  Page 8  Line 209-10 |
| 7. Participant knowledge of the interviewer | What did the participants know about the researcher? | Methods  Page 8  Line 209-12 |
| 8. Interviewer characteristics | What characteristics were reported about the inter viewer/facilitator? | Methods  Page 8  Line 212-14 |
| **Domain 2: study design** |  |  |
| 9. Methodological orientation and Theory | What methodological orientation was stated to underpin the study? | Methods  Page 9  Line 223 |
| 10. Sampling | How were participants selected? | Methods  Page 8  Line 200-01 |
| 11. Method of approach | How were participants approached? | Methods  Page 8  Line 200-01 |
| 12. Sample size | How many participants were in the study? | Methods  Page 8  Line 201 |
| 13. Non-participation | How many people refused to participate or dropped out? Reasons? | Methods  Page 8  Line 199 |
| 14. Setting of data collection | Where was the data collected? | N/A (remote audiorecordings) |
| 15. Presence of non- participants | Was anyone else present besides the participants and researchers? | N/A (remote audiorecordings) |
| 16. Description of sample | What are the important characteristics of the sample? | Methods  Page 8  Line 201, 213-4 |
| 17. Interview guide | Were questions, prompts, guides provided by the authors? | Methods  Page 8  Line 217-220 |
| 18. Repeat interviews | Were repeat interviews carried out? | Methods  Page 8  Line 217-221 |
| 19. Audio/visual recording | Did the research use audio or visual recording to collect the data? | Methods  Page 8  Line 206 |
| 20. Field notes | Were field notes made during and/or after the interview? | N/A (remote audiorecordings) |
| 21. Duration | What was the duration of the interviews | Methods  Page 8  Line 205 |
| 22. Data saturation | Was data saturation discussed? | Methods  Page 8  Line 201-3 |
| 23. Transcripts returned | Were transcripts returned to participants for comment and/or correction? | Methods  Page 9  Line 224-5 |
| **Domain 3: analysis and findings** |  |  |
| 24. Number of data coders | How many data coders coded the data? | Methods  Page 9  Line 223-4 |
| 25. Description of the coding tree | Did authors provide a description of the coding tree? | Methods  Page 9  Line 227-8 |
| 26. Derivation of themes | Were themes identified in advance or derived from the data? | Methods  Page 9  Line 225-7 |
| 27. Software | What software, if applicable, was used to manage the data? | Methods  Page 9  Line 224 |
| 28. Participant checking | Did participants provide feedback on the findings? | Methods  Page 9  Line 224 |
| 29. Quotations presented | Were participant quotations presented to illustrate the themes/findings? Was each quotation identified? | Results  Page 11-19 |
| 30. Data and findings consistent | Was there consistency between the data presented and the findings? | Results  Page 11-19 |
| 31. Clarity of major themes | Were major themes clearly presented in the findings? | Results  Page 11-19 |
| 32. Clarity of minor themes | Is there a description of diverse cases or discussion of minor themes? | Discussion  Page 19-22 |
