# Supplementary material for: Medical student support for vulnerable patients during COVID-19 – a convergent mixed-methods study
Source: BMC Med Educ. 2020 Oct 22;20:377. doi: 10.1186/s12909-020-02305-z (PMC7578590; doi:10.1186/s12909-020-02305-z)
Supplement: Supplementary file 4 — Additional file 4. Interview Questions. [file 12909_2020_2305_MOESM4_ESM.docx]

**Interview questions for students**

1. Tell me about your experience
2. What led you to want to get involved?
3. What were your expectations of the project’s aims before you started?
4. Did your experience differ from this? How?
5. How prepared did you feel at the beginning? How did this change throughout the project?
6. Could you describe any changes to your communication skills/comfort communicating throughout the project?
7. What did you think about the support offered throughout the project?
8. Can you describe any positive experiences you’ve encountered?
9. Can you describe any negative experiences you’ve encountered?
10. Can you describe any experiences that have made you uncomfortable?
11. How well informed do you feel patients were generally before the call?
12. Do you think the calls made through this project offered any specific benefits for patients?
13. How have patient responses changed as the pandemic has progressed? If at all?
14. What do you think/feel patients got out of speaking to you?
15. Were there particular concerns/issues that frequently arose for patients?
16. Did you have any previous experience in discussing End of Life [EoL] care?
17. What was your overall experience in talking about EoL care?
18. How did you feel being asked to ask about EoL care?
19. How receptive did you find people were to thinking about EoL care?
20. Did this change across the duration of the project?
21. Can you describe any positive experiences you’ve encountered relating to EoL discussions?
22. Can you describe any negative experiences you’ve encountered relating to EoL discussions?
23. Can you describe any experiences that have made you uncomfortable relating to EoL discussions?
24. Would you recommend a similar project to other medical students?
25. What advice would you give other medical students thinking of participating in this or similar schemes?
26. What skills (if any) do you think this project might have helped you develop?
27. Could you describe the extent to which you have thought about calls or reflected on conversations after they are complete?
28. Is the anything else you want to share?

**Interview Questions for GPs**

1. What were your experiences of the project?
2. What was your understanding of the project’s aims? Do you think it achieved them?

1. Did you have any concerns regarding medical students calling patients? Did this change throughout the duration of the project?
2. Can you describe any patient responses or reactions you had?
3. Can you describe any experiences of EoL discussions where the patient had previously talked to a med student about this? Do you think the discussion was affected by the earlier conversation?
4. Do you think the project had any impact on overall workload for the practice?
5. How useful do you think the data on how digitally connected patients are?
6. How useful do you think the data on whether or not patients have collected their medications are?
7. How useful do you think the data on permission to pass patient information to support groups are?
8. How do you think this data will be best utilised?
9. How do you think the calls could have been improved? Do you think calls were the best way of getting the information across?

**Interview questions for Academic GP trainee**

1. How did you come up with the idea for this project?
2. What are the aims of this project? Did these evolve during the project?
3. Was the response to your request for volunteers greater or less than you’d expected?
4. Have you had any positive feedback?
5. Have you had any negative feedback?
6. Have you noticed any changes in student attitudes/confidence throughout this project?
7. Has information provided by student phone calls been useful?
8. What role has the scheme played in the surgery’s response to the pandemic?
9. Have you encountered any logistical problems?
10. Which resources did you find most helpful? (e.g. Zoom/Google Drive)
11. What are some difficulties you’ve had in organising this project? How were you able to overcome these?
12. What have you learnt from the project (any take home points)?
13. If you were to run a similar scheme in the future, are there any changes you would make?
14. How did other practitioners respond when you suggested the idea?
15. What was your experience of supporting the students? (Was this a strain? Did you always enjoy it? Were there things you found difficult?)
16. Were there ever times when you were unsure how you should proceed? If so how did you deal with this?
17. What support, if any, was available to you during the course of the project?
18. What concerns did you have when approaching students to have EoL conversations with patients?
19. How did students respond when approached about having EoL conversations with patients?

Did you notice a difference when having EoL **Interview Questions for GPs**

1. What were your experiences of the project?
2. What was your understanding of the project’s aims? Do you think it achieved them?

1. Did you have any concerns regarding medical students calling patients? Did this change throughout the duration of the project?
2. Can you describe any patient responses or reactions you had?
3. Can you describe any experiences of EoL discussions where the patient had previously talked to a med student about this? Do you think the discussion was affected by the earlier conversation?
4. Do you think the project had any impact on overall workload for the practice?
5. How useful do you think the data on how digitally connected patients are?
6. How useful do you think the data on whether or not patients have collected their medications are?
7. How useful do you think the data on permission to pass patient information to support groups are?
8. How do you think this data will be best utilised?
9. How do you think the calls could have been improved? Do you think calls were the best way of getting the information across?

**Interview questions for Emma**

1. How did you come up with the idea for this project?
2. What are the aims of this project? Did these evolve during the project?
3. Was the response to your request for volunteers greater or less than you’d expected?
4. Have you had any positive feedback?
5. Have you had any negative feedback?
6. Have you noticed any changes in student attitudes/confidence throughout this project?
7. Has information provided by student phone calls been useful?
8. What role has the scheme played in the surgery’s response to the pandemic?
9. Have you encountered any logistical problems?
10. Which resources did you find most helpful? (e.g. zoom/ google drive)
11. What are some difficulties you’ve had in organising this project? How were you able to overcome these?
12. What have you learnt from the project (any take home points)
13. If you were to run a similar scheme in the future, are there any changes you would make?
14. How did other practitioners respond when you suggested the idea?
15. What was your experience of supporting the students? (was this a strain, did you always enjoy it, were there things you found difficult).
16. Were there ever times when you were unsure how you should proceed? If so how did you deal with this?
17. What support, if any, was available to you during the course of the project?
18. What concerns did you have when approaching students to have EoLC conversations with patients?
19. How did students respond when approached about having EoLC conversations with patients?
20. Did you notice a difference when having EOLC conversations with patients who had vs hadn’t previously discussed it with a student?
21. What advice would you give to other GPs who are looking to set up a similar project?
22. Do you see a similar type of project being extended for use beyond Covid?
23. Is there anything else you’d like to add?
24. conversations with patients who had vs hadn’t previously discussed it with a student?
25. What advice would you give to other GPs who are looking to set up a similar project?
26. Do you see a similar type of project being extended for use beyond Covid?
27. Is there anything else you’d like to add?
